# Ex-vivo CS1-OKT3 dual specific bivalent antibody-armed effector T cells mediate cellular immunity against multiple myeloma

**DOI:** 10.1038/s41598-023-47115-7

**Published:** 2023-11-27

**Authors:** Dennis Awuah, Lin Li, Lindsay Williams, Ryan Urak, Maciej Kujawski, Stephen J. Forman, John E. Shively, Xiuli Wang

**Affiliations:** 1 T Cell Therapeutics Research Laboratory, Department of Hematology and Hematopoietic Cell Transplantation, City of Hope, Duarte, CA 91010 USA; 2 Department of Immunology and Theranostics, Beckman Research Institute, City of Hope, Comprehensive Cancer Center, Duarte, CA 91010 USA; 3 T Cell Therapeutics Research Laboratory, Beckman Research Institute, City of Hope, Duarte, CA 91010 USA

**Keywords:** Cancer, Immunology

## Abstract

Bispecific T cell engaging antibodies (bsAbs) have emerged as novel and powerful therapeutic agents for redirecting T cells towards antigen-specific tumor killing. The cell surface glycoprotein and SLAM family member, CS1, exhibits stable and high-level expression on malignant plasma cells including multiple myeloma, which is indicative of an ideal target for bsAb therapy. Here, we developed a CS1 bsAb (CS1-dbBiTE) using Click chemistry to conjugate intact anti-CS1 antibody (Elotuzumab) and anti-huOKT3 antibody at their respective hinge regions. Using a cellular therapy approach, human T cells were armed ex-vivo with CS1-dbBiTE prior to examining effector activity. Our data indicates that arming T cells with CS1-dbBiTE induced T cell activation and expansion and subsequent cytotoxic activity against CS1-bearing MM tumors, demonstrated by significant CD107a expression as well as inflammatory cytokine secretion. As expected, CS1-dbBiTE armed T cells showed significantly reduced effector activity in the absence of CS1 expression. Similarly, in MM mouse xenograft studies, armed T cells exhibited effective anti-tumor efficacy highlighted by reduced tumor burden in MM.1S tumor-bearing mice compared to controls. On the basis of these findings, the rationale for CS1 targeting by human T cells armed with CS1-dbBiTE presents a potentially effective therapeutic approach for targeting MM.

## Introduction

Multiple myeloma (MM) is a malignancy of terminally differentiated plasma cells, occurring primarily in the bone marrow (BM) but also in peripheral blood during late-stage disease. It accounts for nearly 2% of all cancers globally and 10% of hematological malignancies in the United States^1^. Although the availability of treatment options (i.e., immunomodulatory drugs, proteasome inhibitors, and monoclonal antibody therapy) has significantly improved the median survival of patients, MM remains largely incurable, requiring renewed efforts toward innovative treatment strategies.

Recently, bispecific T cell engaging antibodies (bsAbs), which combine the specificities of tumor-associated antigens and CD3^+^ T cell targets, have emerged as a novel class of immunotherapy for hematological malignancies including MM^2–5^. By binding to T cells, bsAbs selectively redirect the cytolytic activity of T cells including the release of inflammatory cytokines, perforins and granzymes towards effective tumor cell lysis^6,7^. Additionally, due to their relatively varying sizes, bsAbs offer numerous advantages in terms of production time, dosing regimen and, off-the-shelf provision—advantages currently lacking with current adoptive cell therapies such as CAR T. The relatively small size of bsAbs also confers a limitation, requiring continuous infusions^8^. Indeed, the anti-CD19/CD3 bsAb Blinatumomab, first bsAb approved by the FDA, has shown remarkable success in treating a variety of B cell malignancies including MRD positive and relapsed/refractory B cell-acute lymphoblastic leukemia (B-ALL)^9^, although the therapy proved ineffective in some patients. Nevertheless, given the demonstrated efficacy and potential for safety, bsAbs offer a promising alternative approach for treating malignancies such as MM.

CS1, a cell-surface glycoprotein, and member of the SLAM family, is ubiquitously expressed on malignant plasma cells including MM from patients, with low-level expression on NK and CD8^+^ T cells, and almost undetectable levels on healthy tissues^10–12^. It has been implicated in uncontrolled proliferation and survival of MM cells and circulating CS1 levels are known to correlate with active disease^11,13^. The robust expression of CS1/SLAMF7 on MM cells, including expression on pre-malignant MM stages (i.e., MGUS) makes it an attractive target for therapeutic intervention. Indeed, phase II/III trials with the humanized anti-CS1 antibody (mAb) Elotuzumab (HuLuc63)^11^ have shown clinical efficacy with minimal toxicity^14,15^, suggesting that CS1-targeting lymphocytes do not significantly impact other immune cells. Similarly, CS1-targeting CAR T cells have shown significant promise in our pre-clinical studies, demonstrating efficient cytolytic function and anti-tumor activity in vivo^16^ with tumor relapse occurring after 7 weeks. Clinical trials targeting other MM antigen targets such as BCMA, have also shown incredible response rates^17–19^, however unlike CS1, BCMA and other targets such as CD38 and GPRC5D are not ubiquitously expressed on MM cells, with reports of disease relapse presenting a significant challenge in patient trials. Currently, several bsAbs are in clinical testing against MM^20^, with none against CS1 at the time of writing.

With the aim of targeting plasma cell malignancy in MM that overexpress CS1, we developed a dual specific bivalent bsAb (CS1-dbBiTE) using an efficient Click chemistry method for conjugation of intact anti-CS1 (Elotuzumab) and anti-OKT3 antibodies. Unlike previously reported conjugation procedures with bispecific linkers that conjugated two antibodies via surface lysine residues resulting in a high degree of aggregation^21^, this method dramatically reduces the amount of aggregation, generating bsAbs with improved yields and better biochemical properties. Through our approach of arming T cells prior to co-culture analysis, we emphasize the utilization of the generated CS1-dbBiTE as a modality for cellular therapy. Our findings demonstrate that human T cells armed with CS1-dbBiTEs have the potential to serve as an efficacious therapeutic strategy for targeting MM.

## Results

### Generation of CS1-dbBiTEs and binding to positive targets

**Figure 1:**
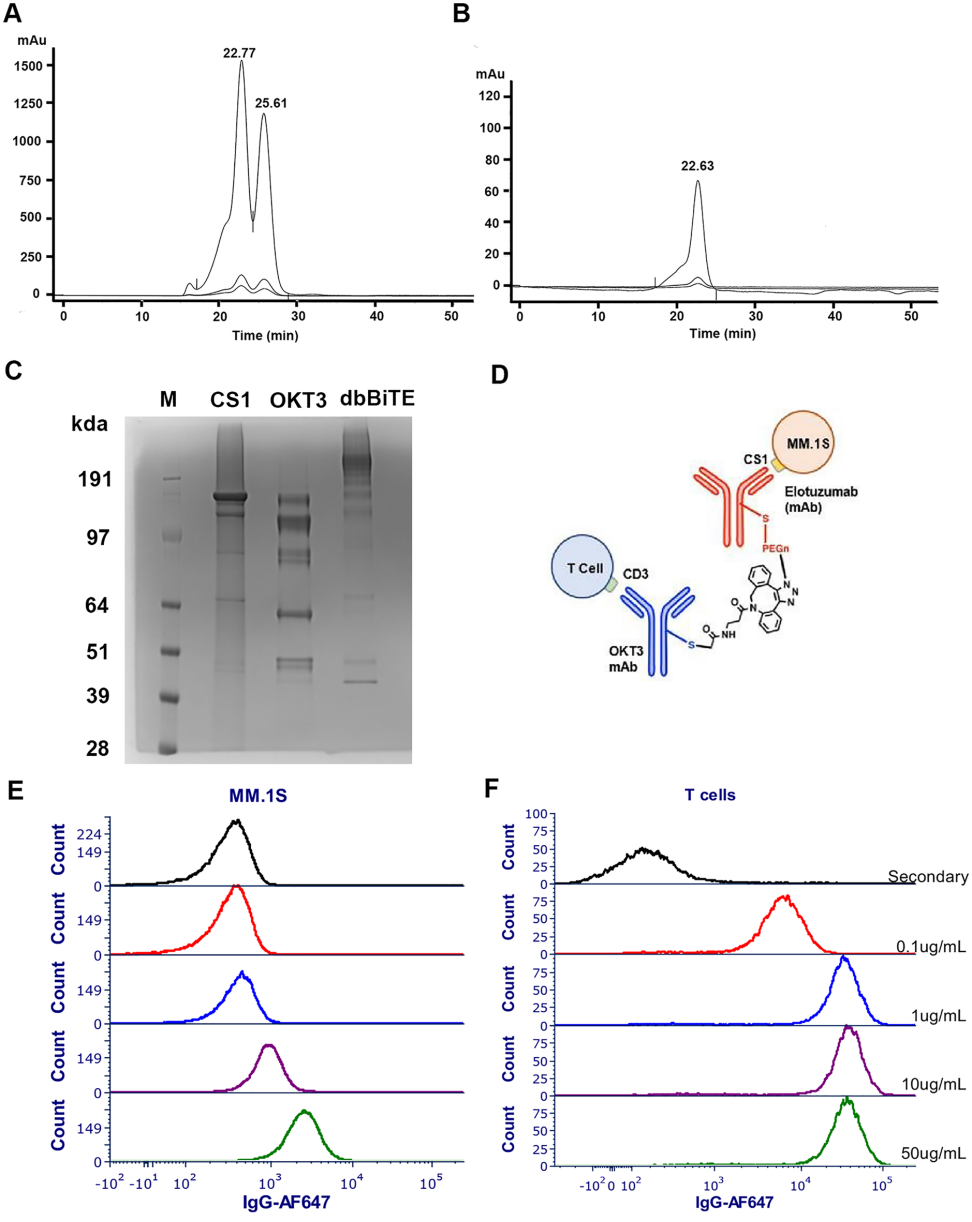
Generation of anti-CS1-huOKT3 dbBiTE and binding to MM.1S and T cells. (**A**) SEC analysis of clicked reaction between the two antibodies shows 2 main peaks, corresponding to the dbBiTE (22.7 min, 300 kDa) and the unreacted antibodies (25.61 min, 150 kDa). (**B**) Re-chromatography of collected dbBiTE peak (22.63 min, 300 kDa). (**C**) Non-reducing SDS gel analysis of individual anti-CS1 and OKT3 antibodies before and after Click chemistry. Gel image is from a single run and not from multiple groupings or cropping. the gel was run longer to increase the resolution at the higher molecular weight range. M; protein marker. Note: huOKT3 runs as intact 150 kDa on SEC, but on SDS gels reveals a portion is lacking the disulfide bridge for one of the heavy-light chains. (**D–F**) Schematic (**D**) and flow analysis of dbBiTE binding to MM.1 s (**E**) and human T cells (**F**) at dilutions as shown in (**F**).

dbBiTEs are bispecific antibodies in which a tumor antigen targeting antibody is crosslinked by Click chemistry to an anti-CD3 antibody^22^. In this study, the starting antibodies were anti-CS1 (Elotuzumab) and a humanized version of anti-CD3 (huOKT3) as previously described^22^. Both antibodies are intact IgG1s that have been modified by Click reagents in their reduced hinge regions. After clicking together, the CS1-dbBiTEs are purified by size exclusion chromatography to separate the unreacted 150 kDa antibodies from their 300 kDa clicked product as shown in Fig. 1A,B. The resulting CS1-dbBiTE exhibits a major band at 300 kDa on non-reducing SDS gels (Fig. 1C). A schematic showing details of the clicked products and their predicted binding to target and T cells is shown in Fig. 1D. Flow analysis of their actual binding to their cognate antigens, namely CS1 on MM.1S and CD3 on human T-cells is shown in (Fig. 1E,F). From this analysis, binding of the CS1-dbBiTE to MM.1S requires a higher concentration than that for binding to human T cells. The differences between the binding are possibly attributed to the varying CS1 density on MM.1S and CD3 density on T cells^11,23^. Thus, further studies were performed at a minimum concentration of 1 µg/mL of CS1-dbBiTE for coating T cells^22^.

### CS1-dbBiTE armed T cells mediate potent cytotoxicity to MM

**Figure 2:**
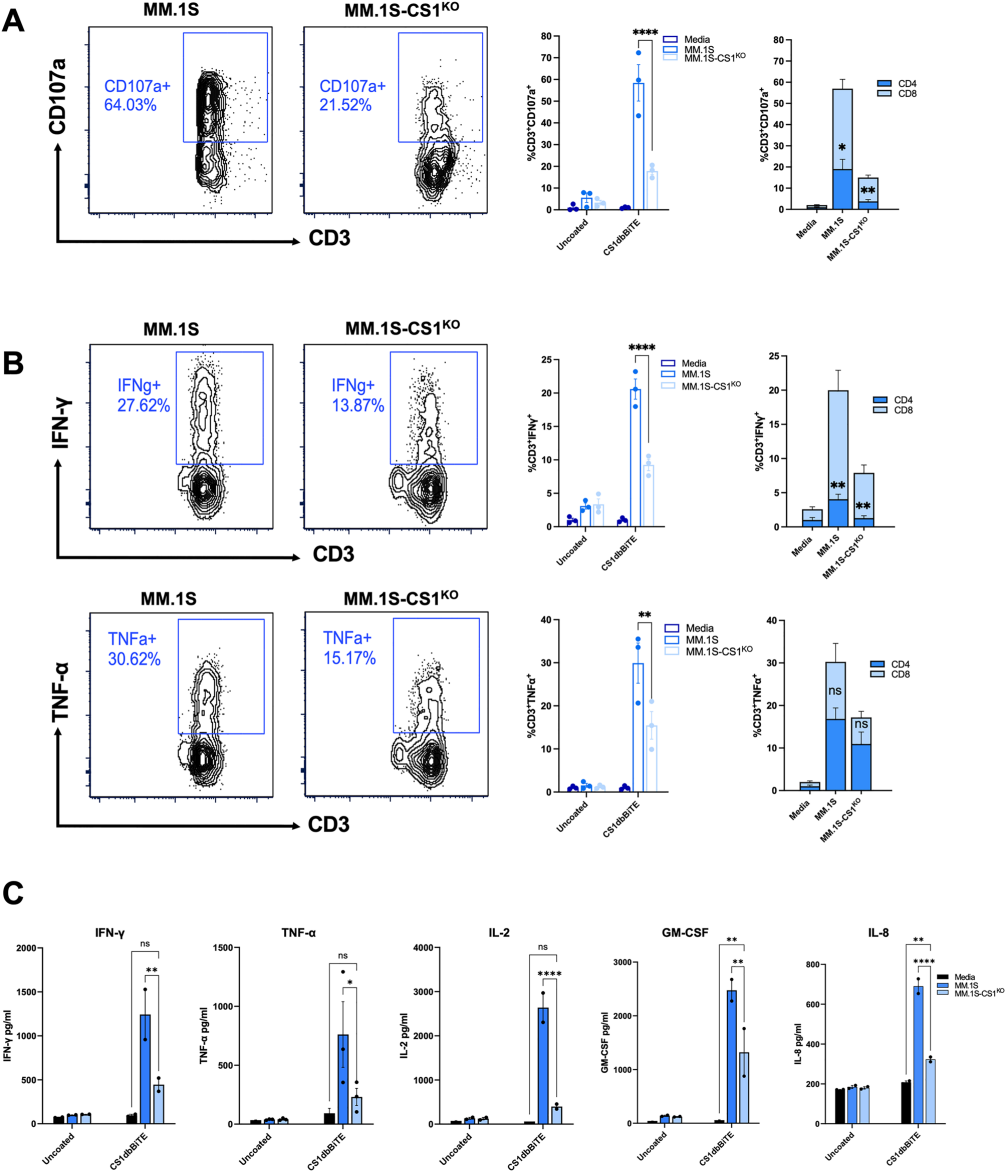
The dbBiTE-armed activated T cells exhibit potent cytotoxic activity: (**A**) 6-h degranulation (CD107a) of dbBiTE-armed, activated T cells following incubation with CS1-expressing or knockout (CS1^KO^) targets. (**B**) Intracellular cytokine staining for IFN-γ and TNF-α expression in CS1-dbBiTE armed T cells after co-culture with tumors. In each case, percentage CD4/CD8 T cell populations are shown. (**C**) Cytokine secretion by dbBiTE-armed, activated T cells in culture supernatants. Representative flow cytometry plots are from single donor (n = 3). Data shows the mean ± SD; P-values determined by two-way ANOVA and Student t test (two-tailed, unpaired). *P < 0.05; **P < 0.01; ***P < 0.001; ****P < 0.0001; *ns* not significant.

T cell engagement with tumor and subsequent activation through CD3 receptor signaling is essential for downstream tumor cell lysis. Following successful CS1-dbBiTE binding to positive targets, we examined the in vitro cytotoxic response of CS1-dbBiTE-armed, activated human T cells (1 µg/mL per 10×10^6^ cells) against multiple myeloma line MM.1S. We chose 1 µg/mL final CS1-dbBiTE concentration based on titration data, and because higher concentrations did not significantly impact effector cell responses (data not shown). In addition, we generated a CS1 knockout MM.1S line (MM.1S-CS1^KO^) to ensure specificity of CS1-dbBiTE binding and found that tumor cell viability was not influenced by CS1 knockout (Fig. S1). As expected, we observed a significant CD107a (*p* = *0.002*) expression by armed T cells against target lines compared to unarmed controls (Fig. 2A). Furthermore, effector cell degranulation was specific for wild-type MM.1S as indicated by a significant reduction in CD107a expression (*P* = *0.0021*) in the absence of CS1-expressing targets. Examination of Th1 cytokine responses as indicators of effective cytolytic activity against tumor cells revealed more than twofold increase in IFN-γ and TNF-α positive cells detected by intracellular cytokine staining (Fig. 2B), when CS1-dbBiTE-armed T cells were co-cultured with MM.1S targets in comparison to MM.1S-CS1^KO^ lines. This observation was consistent with increases in IFN-γ and TNF-α (*P* < *0.05*) production, following analysis of co-culture supernatants (Fig. 2C). The T cell growth factors IL-2 and GM-CSF as well as the chemokine IL-8, were also significantly elevated, whereas no changes were seen in production of Th2 cytokines IL-4, IL-5, IL-6 and IL-10 following target engagement (Fig. S1C). It is noteworthy that degranulating as well as IFN-γ positive cells were predominantly CD8^+^ T cells whereas TNF-α positive cells were primarily CD4^+^ T cells although not significant when compared to CD8 + T cells (Fig. 2A,B).

**Figure 3:**
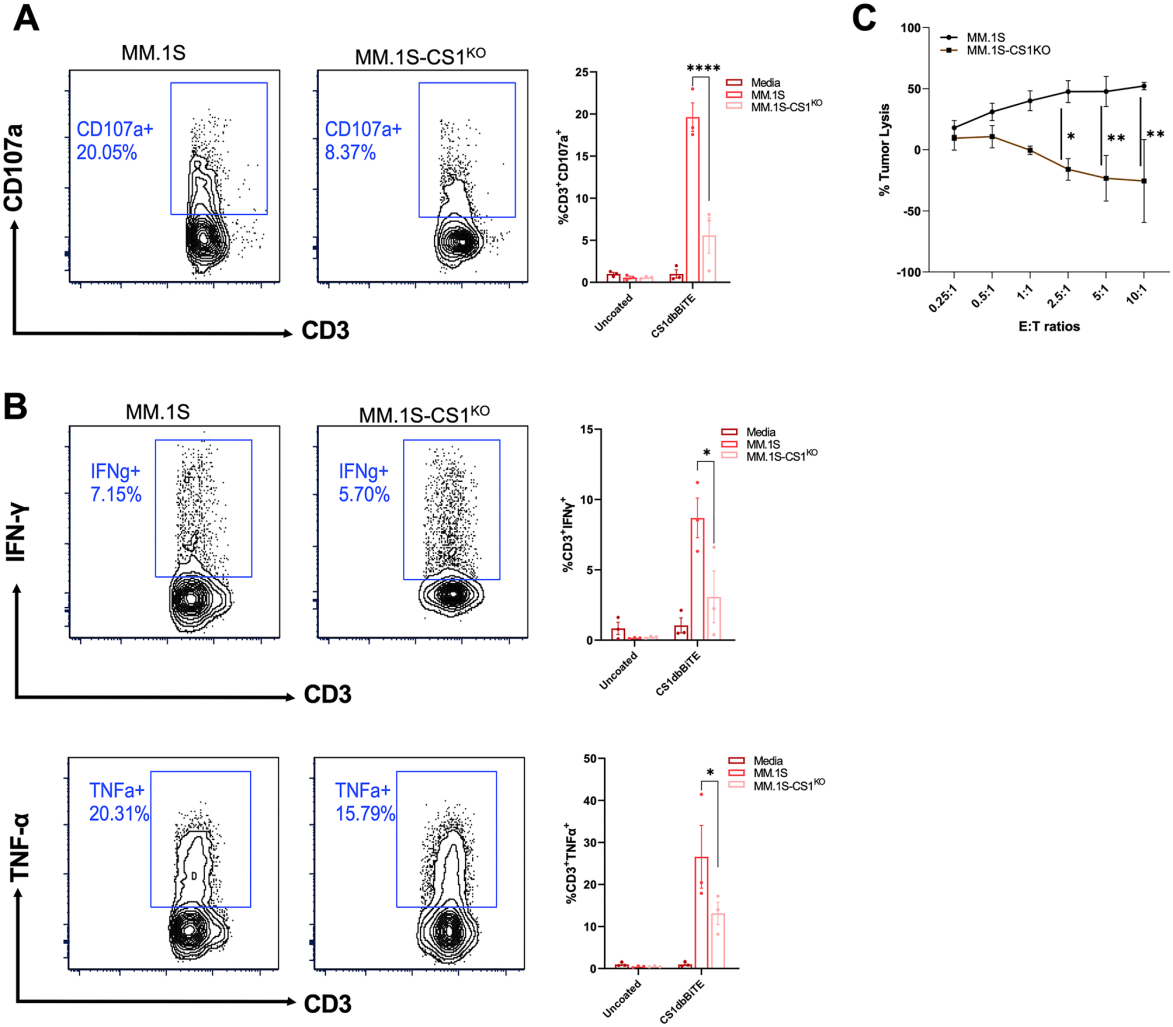
CS1-dbBiTE-armed resting T cells display potent effector activity. (**A**) CD107a expression of dbBiTE armed resting T cells cocultured with MM.1S targets. (**B**) IFN-γ and TNF-α positive T cells following 24-h co-culture with MM.1S and MM.1S-CS1^KO^ target lines (**C**) Long term tumor lysis by armed T cells against specific targets at increasing effector target (e:t) ratios. Representative flow plots are from single donor. 1 μg/mL dbBiTE (per 10×10^6^ cells) was used to generate armed T cells (n = 3). Data shows the mean ± SD; P-values determined by two-way ANOVA. *P < 0.05; **P < 0.01; ****P < 0.0001.

Given that bi-specific T cell engaging antibody therapy is achieved by direct infusion into patients without ex-vivo T cell activation, we also investigated the potency of CS1-dbBiTE armed resting T cells (unstimulated) against target lines (wildtype MM.1S and MM.1S-CS1^KO^). We found that although armed resting cells demonstrated significant CD107a (Fig. 3A) and intracellular cytokine expression (Fig. 3B), the magnitude of effector responses was lower when compared to activated T cells, highlighting the significance of CS1-dbBiTE for cellular therapy approaches as well as potential for direct patient infusion in the clinic. Analysis of long-term killing by armed T cells revealed a dose-dependent lysis of wildtype MM.1S tumors at increasing effector-target (E:T) ratios from 0.25:1 to 10:1, whereas tumor lysis was absent in CS1 knockout lines (Fig. 3C). Similarly, resting T cells also demonstrated dose-dependent tumor lysis with maximum killing (~ 50%) at 10:1 E:T ratio (Fig. S2). In contrast to activated T cells, degranulating and intracellular cytokine positive, resting T cells were predominantly CD4^+^ cells (Fig. S2B).

### CS1-dbBiTE induces T cell activation and differentiation

**Figure 4:**
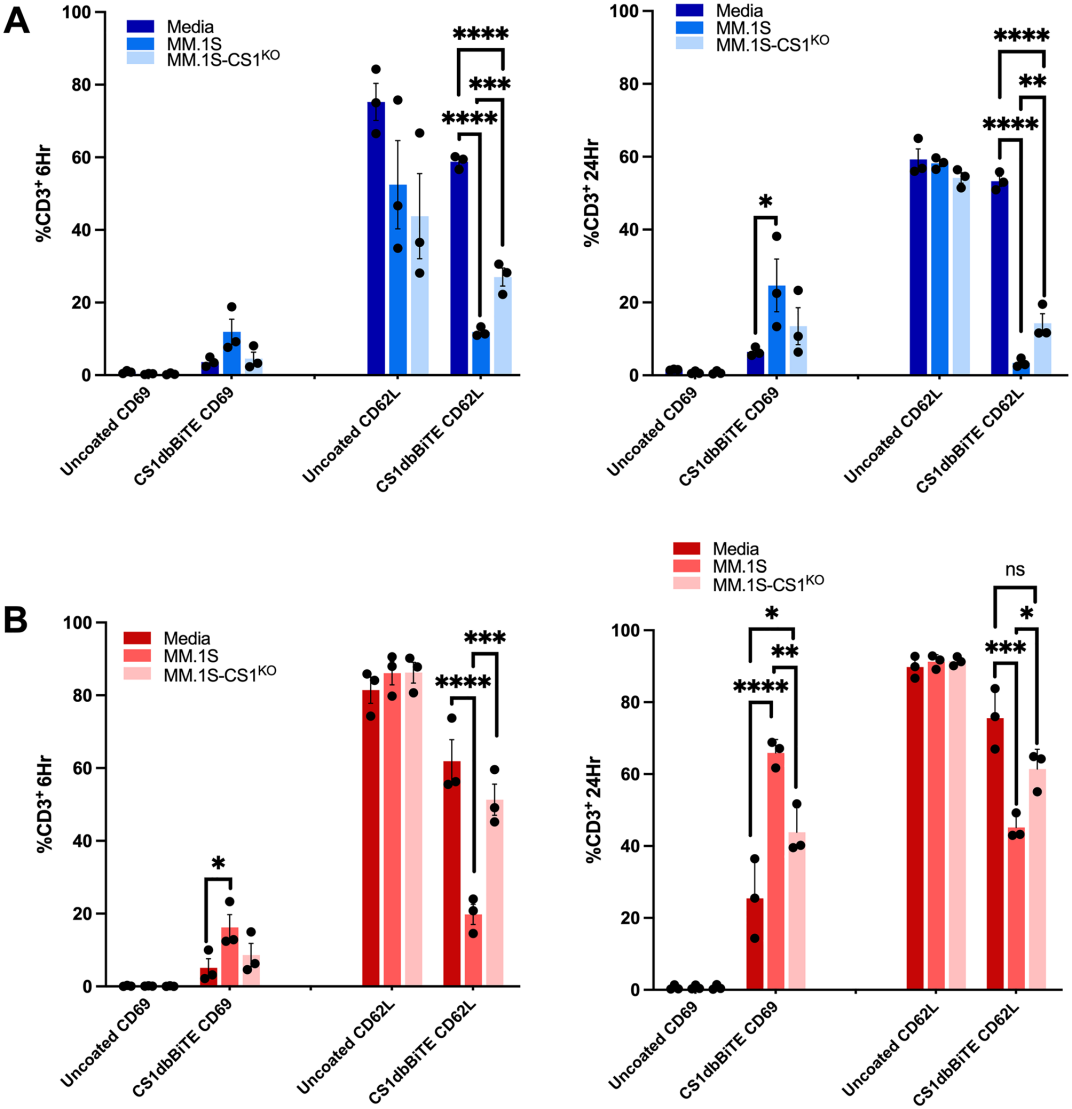
CS1-dbBiTE promotes T cell activation and differentiation. Expression of CD69 activation and CD62L memory markers after 6 and 24 h respectively between dbBiTE-armed (**A**) activated and (**B**) resting T cells, and subsequent incubation with CS1 positive targets (n = 3). Data shows the mean ± SD; P-values determined by Student t test (two-tailed, unpaired). *P < 0.05; **P < 0.01; ***P < 0.001; ****P < 0.0001; *ns* not significant.

We further examined whether arming T cells with CS1-dbBiTE alone (in the absence of antigen) influenced T cell activation and memory marker expression and found an increase in expression of the early activation marker CD69, above baseline levels in both CS1-dbBiTE-armed, activated (Fig. 4A) and resting T cells (Fig. 4B) suggesting that arming T cells with CS1-dbBiTE only may promote T cell activation. Additionally, we observed a significantly higher CD69 expression in the presence of CS1 targets (antigen) after 24 h of co-culture, with resting cells demonstrating greater than 40% increase in CD69 expression compared to ~ 10% increase in activated cells, which could presumably reflect the different activation states after two activation cycles (activated) vs. one activation (un-stimulated) cells. The absence of CD69 expression in unarmed but activated cells after 6- and 24-h, following CD3/CD28 stimulation suggests timing is crucial for expression of CD69. Conversely, CD62L memory marker showed high expression in unarmed cells but decreased expression in the presence of CS1 targets following CS1-dbBiTE arming (Fig. 4A,B), highlighting the differentiation of T cells from a more central memory phenotype to effector memory populations.

**Figure 5:**
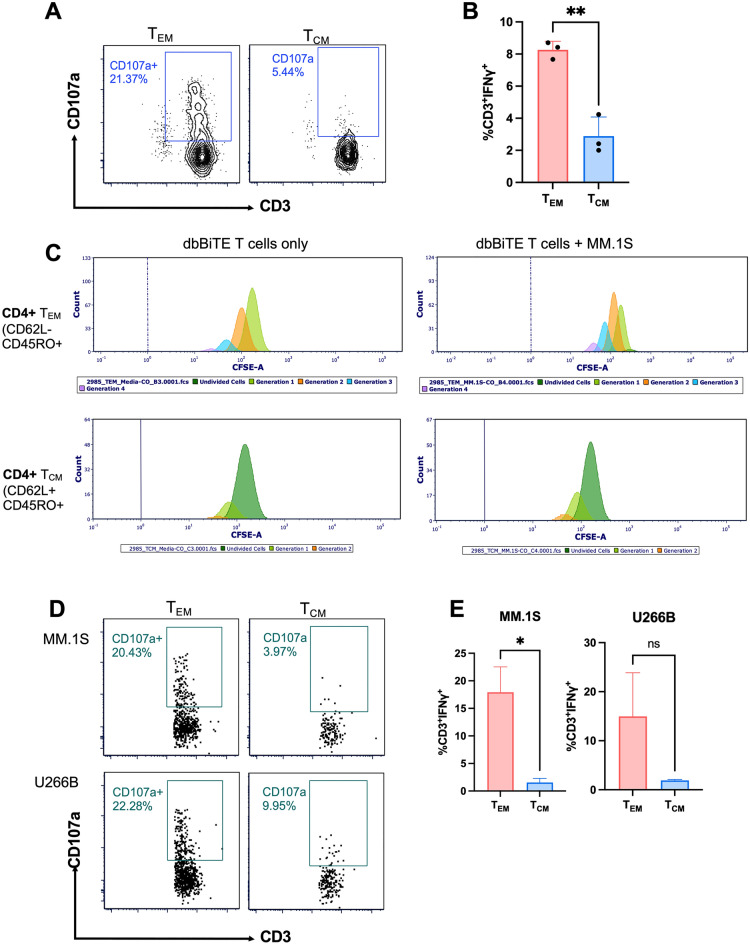
Effector memory but not central memory dbBiTE-armed T cells mediate specific effector function. Comparison of dbBiTE mediated (**A**) degranulation and (**B**) IFN-γ production between effector memory (T_EM_) and central memory (T_CM_) T cells following co-culture with MM.1S. (**C**) T_EM_ cells exhibit superior proliferative capacity compared to TCM in the presence of dbBiTE alone or in the presence of CS1-expressing MM.1S target cells. Effector memory cells (T_EM_) from patient samples armed with CS1-dbBiTEs display superior. Color represents the generations of cell division. (**D**) CD107a expression and (**E**) IFN-γ positive cells, when co-cultured with multiple myeloma target lines MM.1S and U266B. Representative plots are from a single donor (n = 3). Data shows the mean ± SD; P-values determined by two-way ANOVA. *P < 0.05; **P < 0.01; *ns* not significant.

In line with our observations, previous studies in patients receiving the BiTE antibody, blinatumomab for treatment of non-Hodgkin’s lymphoma (NHL) have demonstrated fold increases in T cell numbers over baseline levels, with T cell expansion levels prominent in effector memory (T_EM_) subset^24^. T cell fitness in patients could vary due to multiple lines of treatment and intrinsic dysfunction of T cells. To determine the impact of T cell differentiation state on CS1-dbBiTE on activity, we performed T cell subset isolation from healthy donor PBMC and evaluated their cytotoxic and proliferative responses against MM following CS1-dbBiTE arming. T cell proliferation was determined using CellTrace™ CFSE staining. We found that CD62L-CD45RA- T_EM_ cells induced significant CD107a expression and were positive for IFN-γ expression when co-cultured with MM.1S lines, whereas no cytotoxic activity was observed for CD62L^+^CD45RA^+^ central memory (T_CM_) T cells (Fig. 5A,B). Moreover, T_EM_ cells exhibited superior proliferative capacity in the presence of CS1-dbBiTE alone or CS1-dbBiTE with CS1 targets after 5 days of co-culture compared to T_CM_ cells (Fig. 5C). Interestingly, proliferating cells from the T_EM_ subset were mainly CD4 T cells but not CD8 T cells (Fig. S3A). Examination of CS1-dbBiTE-armed CD62L-CD45RA- PBMCs from MM patient samples also revealed significant CD107a (Fig. 5D) as well as IFN-γ expression following co-culture with the CS1 positive targets, MM.1S and U266B (Fig. 5E; Fig. S3B), highlighting the potential of our CS1-dbBiTE treatment against MM. Similar to T_CM_ cells, CD62L^+^CD45RA^+^ stem cell memory (Tscm) T cells from healthy donors did not induce CD107a expression in the presence of MM.1S during co-culture (Fig. S3C). Thus, our data suggests that effector memory T cells are the dominant subsets for CS1-dbBiTE mediated effector function in patients and differential proportions of T_EM_ in patients may contribute to varying responses to CS1-dbBiTE infusion clinically. Gating strategy for flow data is shown in Fig. S4.

### Anti-tumor efficacy of CS1-dbBiTE armed T cells

**Figure 6:**
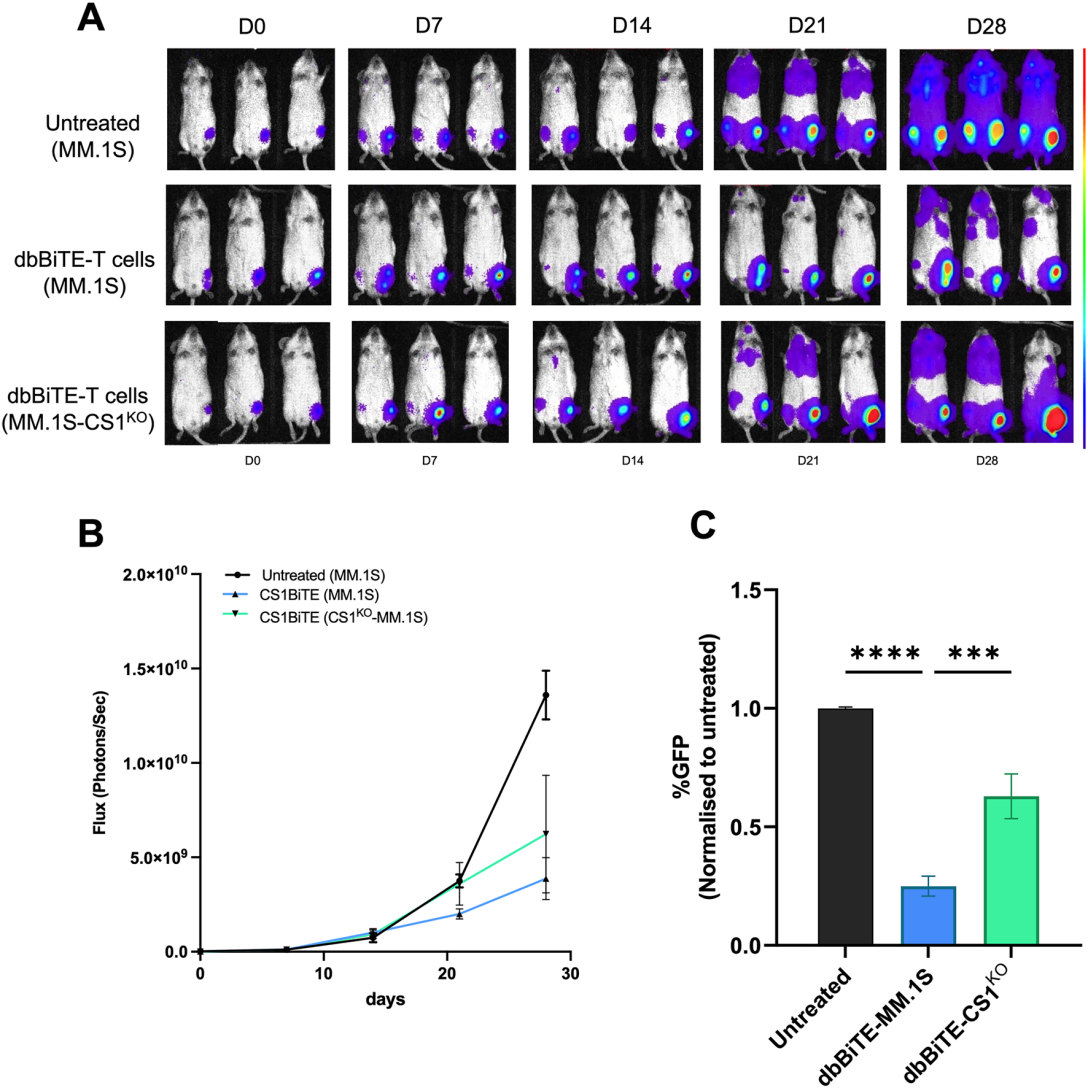
Anti-tumor activity of dbBiTE-armed T cells in vivo. (**A**) Tumor progression in mice depicted by bioluminescence imaging (BLI) following engraftment and weekly dbBiTE-T cell infusions. 5 mice per group. Representative mice are presented. (**B**) Comparison of flux curves (Photons/Sec) between untreated and CS1-dbBiTE-T cell treated conditions. (**C**) Residual GFP^+^ tumor cells in bone marrow following tissue harvest from respective conditions shown. MM.1S or MM.1S-CS1^KO^ cells were engrafted by intra-tibial injection on day 0 and tumor progression was monitored weekly for 5 weeks. ***P < 0.001; ****P < 0.0001.

Given the demonstrated efficient cytotoxicity against MM, and cytokine production profile of our CS1-dbBiTE armed T cells in vitro, we hypothesized that armed T cells would possess superior anti-tumor efficacy against wildtype MM.1S tumor bearing mice over CS1-knockout MM xenograft mice. To this end, we engrafted NSG mice with 2 × 10^6^ MM.1S-eGFP or MM.1S-CS1^KO^-eGFP lines via intra-tibial injections and administered PBS or 5 × 10^6^ CS1-dbBiTE-armed T cells intravenously, 6 days post tumor engraftment. Treatments were administered once weekly for 4 weeks, and tumor burden was monitored weekly by bioluminescence imaging for 5 weeks (Fig. 6A). As shown in Fig. 6, MM.1S-eGFP xenograft mice that received CS1-dbBiTE-armed T cells were able to control tumor burden 4–5 weeks post treatment depicted by a reduction in flux compared to untreated mice and MM.1S-CS1^KO^ engrafted mice treated with CS1-dbBiTE (Fig. 6B). Examination of mice bone marrow (BM) samples also revealed similar patterns of residual tumor cells (GFP +), with untreated and MM.1S-CS1^KO^-eGFP xenograft mice showing significantly higher GFP^+^ cells compared to MM.1S-eGFP engrafted mice (Fig. 6C). Although 5 × 10^6^ CS1-dbBiTE armed T cells were engrafted weekly into treatment groups, we did not detect circulating T cells in mouse tissues (blood, spleen, and BM) post tissue harvest.

## Discussion

In this study, we assessed the pre-clinical efficacy of human T cells armed with newly generated CS1-dbBiTEs against multiple myeloma and observed increased T cell activation and expansion as well as significant effector function including production of inflammatory cytokines and in vitro tumor lysis. Conversely, these observations were absent in unarmed T cells and significantly downregulated during co-culture with CS1 knockout MM targets. Traditionally, CD3-engaging bsAbs comprise two genetically engineered single chain variable fragments (scFv), fused by a flexible linker. Alternatively, we cross-linked two intact antibodies to generate antibody molecules of dual specificities with bivalent binding (dbBiTE)^22^, circumventing the need to engineer bsAbs that may be difficult to properly fold or express, with poor pharmacokinetic properties when administered in patients.

Elotuzumab is an FDA approved monoclonal antibody for treatment of MM, which exhibits a dual mechanism of action through direct NK cell activation when bound to SLAMF7 on its surface or by antibody dependent cellular cytotoxicity (ADCC)^25,26^. Additional modes of action have also been reported via other SLAMF7 positive cells including dendritic cells and CD8^+^ T cells^27^. Nevertheless, due to its suboptimal efficacy in patient trials, Elotuzumab is used in combination with immunomodulatory drugs and steroids such as lenalidomide and dexamethasone to achieve better efficacy^15,28,29^. By conjugating intact Elotuzumab and humanized anti-OKT3 antibodies, we sought not only to improve the efficacy of Elotuzumab but also benefit from its safety profile, which has been consistent across multiple clinical studies. Additionally, arming T cells ensured that the right binding affinity/ biological activity of the fused molecule could be determined, allowing for removal of excess dbBiTEs. The relatively larger size of the CS1-dbBiTE (~ 300 kDa) molecule is also likely to confer better pharmacokinetic properties in patients. In contrast, anti-CD3 antibodies such as OKT3 or UCHT1 antibodies can promote cross-linking of the TCR signaling complex leading to non-specific activation of T cells in the absence of antigen, ultimately resulting in substantial release of inflammatory cytokines and soluble mediators^30,31^. Indeed, IL-6 and TNF-α have been implicated as central mediators of cytokine release syndrome (CRS) a major toxicity associated with T cell immunotherapy^32^. For instance, clinical administration of an anti-CD28 antibody to healthy individuals in a phase one study resulted in peak levels of inflammatory cytokines TNF-α, IL-2, -6 and -10 as well as IFN-γ, and IL-1β within the first few hours of infusion^33^. In this study, arming T cells with CS1-dbBiTEs did not promote cytokine release in the absence of antigen suggesting the need for antigen dependency.

Conversely, clinical administration of T cells armed with bsAbs have been shown to lack indicators of CRS^34,35^ highlighting their safety. In line with these studies, we observed significantly lower IFN-γ or TNF-α positive cells as well as lower cytokine secretion from CS1-dbBiTE-armed T cells alone, with cytokine levels peaking only in the presence of antigen. Furthermore, considering the cell-based therapeutic approach, only microgram (µg) amounts of CS1-dbBiTEs are needed for T cell arming, thus limiting the occurrence of non-specific activation and cytokine release. In addition to utilizing FDA approved therapeutic antibodies for dbBiTE development, the use of µg concentrations can be achieved without further regulatory concerns. Such an approach could potentially provide ready-to-use antigen-specific T cells that may be used in combination with other cellular therapies such as CAR T cells for treatment of a variety of hematologic malignancies.

Although bsAbs and CAR T cells are at the forefront of cell-mediated cancer therapies, bsAbs possess numerous advantages over CAR T cells in the clinic, including immediate availability to patients. The short half-life also ensures that bsAb therapy can be halted at any time upon discovery of safety-related issues such as on target/off tumor effects. The inability to obtain sufficient T cells from some patients due to poor ‘fitness’ also implies that certain candidates remain ineligible for CAR T therapy but may still be able to obtain bsAb therapy, since it is likely they will maintain effector T cell populations with the capacity to respond rapidly to bsAb treatment^36,37^. In line with previous studies^24^, our data demonstrates that effector memory T cell populations, which likely require less stimulation to be fully activated, are the more responsive subsets (compared to central memory T cells) to cell proliferation and subsequent effector function when armed with CS1-dbBiTE. The mechanism of bsAb tumor killing on the other hand, is reported to be similar to that of CAR T cells, involving initial synapse formation and subsequent release of soluble mediators such as perforins and granzymes. Nevertheless, given that bsAbs engage the natural CD3 complex, (whereas CARs do not) the synapse that forms between T cells and tumor targets when linked by bsAbs likely resembles synapses formed during physiologic T cell antigen recognition^38,39^, leading to increased proliferation and upregulation of activation markers such as CD69 as demonstrated in this study. Therefore, although bsAbs are used by continually infusing into patients for better therapeutic efficacy, we find that CS1-dbBiTE-armed T cells also exhibit significantly potent effector function either in the presence or absence of in vitro bead activation, suggesting that (1) banked T cells (bead stimulated or not) can be obtained and armed with CS1-dbBiTE at any point when needed and (2) CS1-dbBiTEs could potentially be infused directly into patients to still achieve the desired clinical effect.

An advantage of CAR T over bsAb therapy, however, is the potential for CAR T cells to engraft long-term in vivo, providing a source of antigen-experienced T cells capable of responding to recurring tumors^39,40^. In other studies, although bsAbs were administered in xenograft mice once daily for 5 days, the short half-life meant bsAbs were not present at later stages of tumor progression, even though it is likely to have delayed metastasis^41^. Similarly, studies in MM xenograft mice treated with novel BCMA/CD3 bsAbs after intraperitoneal transfer of ex-vivo expanded T cells also revealed that transferred T cells failed to engraft in non-responding mice following examination of tissues samples and were > 98% positive for BCMA expression^42^. In line with these observations, we found that MM.1S xenograft mice receiving CS1-dbBiTE armed T cells (once weekly for 4 weeks) were able to reduce tumor burden compared to MM.1S-CS1^KO^ xenograft mice and untreated controls, however armed cells failed to engraft long term and were undetectable in mouse tissues (blood, spleen, and marrow) following analysis. Interestingly, we did not observe antigen loss in the relapsed tumor. However, it is worth noting that the same concentration of CS1-dbBiTE for both in vitro and in vivo experiments were used. It is reasonable to consider that a higher concentration of CS1-dbBiTE may be required to coat T cells for in vivo experiments to achieve better potency or more frequency of in vivo infusion instead of once a week. Considering the reduction in tumor burden observed, it is likely CS1-dbBiTE armed T cells may have reduced tumor metastasis and perhaps if followed longer, may have been able to prolong mouse survival.

In summary, this study has demonstrated an efficient approach for targeting MM tumors by utilizing intact and clinically available antibodies (Elotuzumab and OKT3) fused to generate a bispecific molecule for redirecting T cells towards CS1-expressing MM killing. Although dbBiTEs are still in developmental stages, and applicable to a variety of therapeutic antibodies, our findings support the use of CS1-dbBiTEs as an effective therapeutic approach for targeting MM. Through the pre-complexed arming approach, dbBiTE has the potential to be used in combination cellular therapy targeting different antigens. This approach could offer the advantage of producing activated T cells as an additional side product during the manufacturing process, along with other cellular products derived from the same patients.

## Materials and methods

### Ethics declaration

All animal studies were performed in accordance with IACUC protocol approved by the City of Hope Institutional Animal Care and Use Committee (IACUC 21034), and in accordance with the National Institutes of Health (NIH) Office of Laboratory Animal Welfare guidelines. Animal data generated was reported according to ARRIVE guidelines.

### Generation of CS1-dbBiTE

CS1 (2 mg, 13.33 nmol) in 200 µl of PBS was reduced with a 30-molar excess of tris (2-carboxyethyl) phosphine (TCEP) and 1 mM of EDTA at 37 °C for 2 h under Argon. The TCEP was removed by using a desalting spin column (Zeba, 7KDa MW cutoff, Thermo Fisher Scientific, Waltham, MA). The reduced CS1 was reacted with a 20-fold molar excess of bromoacetamido-DBCO (Broadpharm, San Diego, CA) in pH 7.4 at RT overnight under Argon. The excess bromoacetamido-DBCO was removed by dialysis in PBS (2Lx5). The conjugation was confirmed by Agilent 6520 QTOF mass spectrometry. The light chain had one DBCO/light chain and the heavy chain an average of 3 DBCO/heavy chain. Anti-hu-OKT3 has been previously described^22^. Anti-hu-OKT-3 (5 mg, 33.33 nmol) in 858 µl of PBS was reduced with a 30-molar excess of tris (2-carboxyethyl) phosphine (TCEP) and 1 mM of EDTA at 37 °C for 2 h under Argon. The TCEP was removed by using a desalting spin column (Zeba, 7KDa MW cutoff). The reduced anti-hu-OKT-3 was reacted with a 60-fold molar excess of bromoacetamide-PEG_5_-N3 (Broadpharm, San Diego, CA) in pH 7.4 at RT overnight under Argon. The excess bromoacetamide-PEG_5_-N3 was removed by dialysis in PBS (2Lx5). The conjugation was confirmed by Agilent 6520 QTOF mass spectrometry. The light chain had one PEG_5_-N_3_ and heavy chain an average of 3 PEG_5_-N_3_/heavy chain. CS1-DBCO (1.65 mg, 11.0 nmol) in 500 µl PBS was incubated with anti-hu-OKT3-PEG_5_-N_3_ (1.65 mg, 11.0 nmol) in 580 µl PBS, pH 7.4 at RT for 2 days under Argon. The clicked antibodies were purified by size exclusion chromatography on a Superdex 200, 10 × 300 GL column (GE Healthcare) at a flow rate of 0.5 mL/min in PBS using a GE AKTA Purifier. The 300 kDa peak was collected and concentrated to 2.93 mg/mL.

### Antibodies and flow cytometry

Mouse monoclonal antibodies against human CD3, CD4, CD8, CD69, CD62L CD45RA, CD45RO, CD25, CD62L, CD107a were obtained from BD Biosciences. The anti-CS1 antibody was obtained from R&D Systems. Briefly, cells were harvested and washed twice in FSS solution (PBS buffer containing 2% FCS and 0.5% NAN_3_). Staining with labeled antibodies was then carried out in the dark at 4˚C for 15 min according to manufacturer’s protocol. Unless otherwise stated, antibodies were fluorochrome conjugated to APC, APC/Cy7, FITC, PE, or PE/Cy7, PerCP or Viogreen (Brilliant violet 510). Following staining, samples were washed twice with FSS solution before analysis. Cell viability was determined using Dapi staining. Nonreactive, isotype-matched antibodies were used as controls. Flow cytometry was carried out on MACSQuant (Miltenyi Biotec), and data analysis was performed with FCS Express Version 7 (De Novo Software) for Windows.

### Cell lines

The multiple myeloma line MM.1S was purchased from ATCC and cultured in RPMI media supplemented with 10% heat inactivated FBS. To generate firefly luciferase^+^ GFP^+^ MM.1S (eGFP^+^ffluc^+^), MM.1S cells were transduced with lentiviral vector encoding eGFP-ffluc; transduced cells were further sorted by FACS to obtain > 98% purity. MM.1S-CS1^KO^ cells were generated by CRISPR/Cas9 system^43^ using appropriate guide RNAs (gRNA) against SLAMF7, and knockout population were sorted using anti-CS1 antibody (R&D Systems). Prior to cryopreservation, cell lines were authenticated for expression (or lack) of desired surface markers by flow cytometry and thawed cells were cultured for 3—6 weeks prior to use in assays.

### T cell isolation and activation

Leukapheresis products were obtained from healthy donors, and peripheral blood mononuclear cells (PBMCs) were separated by density gradient centrifugation on Ficoll-paque (Amersham Biosciences). T naïve/memory (Tn/mem) cells were isolated by autoMACS (Miltenyi Biotec) using CD62L^+^ magnetic beads following depletion of CD14^+^ and CD25^+^ cell fractions, and resulting cells were activated with CD3/CD28 microbeads (Invitrogen) as previously described^44,45^. In some experiments, healthy donor PBMCs were FACS sorted for central memory (Tcm), effector memory (Tem) and stem cell memory (Tscm) T cells without CD3/CD28 bead stimulation. All healthy donor samples were obtained under approved City of Hope Institutional Review Board (COH IRB) protocols (IRB09025) in accordance with the Declaration of Helsinki.

### Coating conditions for CS1-dbBiTE

Target cells (MM.1S and activated CD3^+^ Tn/mem cells) were initially incubated in 1% goat serum in PBS for 30 min and subsequently incubated on ice with increasing concentrations of CS1-dbBiTE antibody (0.1 ug/mL–50 ug/mL per 10 × 10^6^ cells) for 15–30 min. In some experiments, incubation was carried out for 1 h. Cells were then washed twice to eliminate excess unbound dbBiTE and labelled with 2 ug/mL goat anti-mouse Alexa647 or goat anti-human Alexa555 secondary antibodies (Thermofisher Scientific) for detection of bound CS1-dbBiTE. Following wash steps, cells were resuspended in PBS and analyzed by flow cytometry on MACSQuant (Miltenyi). Unlabeled dbBiTE cells or cells labeled with secondary antibodies only were used as controls. In co-culture experiments, T cells (in complete RPMI media) were armed with dbBiTE for 15 min at room temperature, washed twice with plain RPMI media before use in assay.

### Degranulation assay

Degranulation assay was done as previously described^44^. Briefly, CS1-dbBiTE-armed T cells or PBMCs were co-cultured with MM.1S and MM.1S-CS1^KO^ target lines at an effector to target (E:T) ratio of 2:1 in complete RPMI medium containing Golgi Stop solution (BD Biosciences). Anti-CD107a antibody was added to each well and the co-culture setup was incubated for 5–6 h at 37 °C. Unarmed T cells were used as controls. Degranulation was assessed by multicolor flow cytometry on MACSQuant analyzer (Miltenyi).

### Intracellular cytokine staining

Target cells (0.2 × 10^6^) were plated in 96-well plates and co-cultured with 0.2 × 10^6^ CS1-dbBiTE-armed CD3^+^ T cells for 4 h at 37 °C in RPMI media, supplemented with 10% FBS. Golgi plug media (brefeldin A; BD Biosciences) was subsequently added to each well and incubated overnight at 37 °C. Cells were washed and stained for surface marker detection prior to intracellular staining. Following fixation and permeabilization, (cytoperm kit; BD Biosciences), cells were stained with anti-IFNγ and anti-TNFα antibodies (BD Biosciences) for 30 min at 4 °C and washed twice with perm wash (BD Biosciences) before analysis on MACSQuant analyzer (Miltenyi). Unarmed T cells were used as control.

### Cytokine analysis

CS1-dbBiTE armed T cells were co-cultured at 1:1 E:T ratio with MM.1S or MM.1S-CS1^KO^ target cells overnight in 96-well plates. The culture supernatant was collected and stored at -20 °C until analysis. Cytokines were measured with the Human Cytokine Magnetic Bead Array (10-plex), according to the manufacturer's instructions (Invitrogen).

### In vitro cytotoxicity assay

Cytotoxicity assay was carried out as previously described. Briefly, eGFP-ffluc-expressing MM.1S and MM.1S-CS1^KO^ target cells were plated in 96-well plates at a concentration of 0.1 × 10^6^ per well in 100μL of RPMI media containing 10% FBS. CS1-dbBiTE-armed, resting and activated T cells were then added at varying concentrations to final E:T ratios of 0.25:1, 0.5:1, 1:1, 2.5:1, 5:1 and 10:1, respectively. In both conditions, unarmed T cells were used as controls and plated according to same E:T ratios. Co-culture cells were incubated for 24 h at 37 °C and analyzed by flow cytometry. Tumor killing was determined by percentage expression of eGFP-ffluc^+^ cells.

### CFSE proliferation assay

Tumor cells (0.1 × 10^6^) were co-cultured in RPMI medium for 96 h with CS1-dbBiTE armed, central (CD62L^+^CD45RA^+^) and effector memory (CD62L-CD45RA-) T cells at 1:1 E:T ratio. Prior to co-culture, armed T cells were labelled with or without 5 µM CellTrace CFSE (Thermo Fisher Scientific) and incubated for 20 min at 37 °C. Thereafter, cells were harvested and stained with mouse anti-human CD3, CD4 and CD8 antibodies before analysis. T cell proliferation by the different subsets was determined by flow cytometry on MACSQuant.

### Xenograft models

All mouse experiments were approved by the City of Hope Institutional Animal Care and Use Committee (COH-IACUC21034) and were performed in NOD.Cg-Prkdcscid Il2rgtm1Wjl/SzJ mice (NSG; 6–10 weeks old). Mice were kept in pie cages in a specific pathogen free (SPF) room, with a maximum of 5 mice per cage. NOD/SCID IL2RgCnull (NSG) mice were engrafted on day 0 with 2 × 10^6^ eGFP^+^ffluc^+^ MM.1S or MM.1S-CS1^KO^ target cells via intra-tibial delivery (i.t). Mice were imaged and sorted on day five following tumor engraftment, and subsequently treated next day with 5 × 10^6^ unarmed or CS1-dbBiTE-armed T cells intravenously (i.v). Infusions with armed or unarmed T cells (5 × 10^6^/mouse) were carried out once a week for 4 weeks. Effector T cells were armed with 1 ug/mL CS1-dbBiTE per 10 × 10^6^ cells. Tumor burden was monitored once weekly by bioluminescence imaging using Xenogen imager (Spectral Instruments Imaging). Mice were sacrificed after 5 weeks post-tumor engraftment and tissue samples were harvested for analysis by flow cytometry. Mice were checked on daily basis by resident veterinarian to ensure proper animal health and welfare, and recommendations for mice euthanization was issued following discovery of distressed mice. Euthanasia was carried out using 100% carbon dioxide (CO_2_) in an euthanasia chamber (at a fill rate of 30–70% of chamber volume per minute) before cervical dislocation and tissue collection.

### Statistical analysis

Data was analyzed using GraphPad Prism version 9 for Windows (GraphPad Software, San Diego, CA), and values expressed as mean ± SD from independent experiments unless otherwise stated. Paired t tests or ordinary one-way ANOVA was used when comparing between two groups or three or more groups, respectively. For animal studies, Kaplan–Meier survival analysis was performed, and statistical significance was calculated using log-rank (Mantel-Cox). A p value ≤ 0.05 was considered statistically significant.

### Supplementary Information

Supplementary Figures.

## Data Availability

All data associated with this study are present in the paper or can be found in the provided supplemental information.
