# Supplementary material for: Ex-vivo CS1-OKT3 dual specific bivalent antibody-armed effector T cells mediate cellular immunity against multiple myeloma
Source: Sci Rep. 2023 Nov 27;13:20853. doi: 10.1038/s41598-023-47115-7 (PMC10682018; doi:10.1038/s41598-023-47115-7)
Supplement: Supplementary file 1 — Supplementary Figures. [file 41598_2023_47115_MOESM1_ESM.docx]

**Supplemental Information**

**Ex-vivo CS1-OKT3 Dual Specific Bivalent Antibody-Armed Effector T Cells Mediate Cellular Immunity against Multiple Myeloma**

Dennis Awuah^1^, Lin Li^2^, Lindsay Williams^2^, Ryan Urak^1^, Maciej Kujawski^2^, Stephen J. Forman^1^, John E. Shively^2^, and Xiuli Wang^1^*

^1^ T Cell Therapeutics Research Laboratory, Department of Hematology and Hematopoietic Cell Transplantation, City of Hope, Duarte, CA, 91010

Beckman Research Institute, City of Hope, Comprehensive Cancer Center, Duarte, CA, 91010.

^2^ Department of Immunology and Theranostics, Beckman Research Institute, City of Hope, Comprehensive Cancer Center, Duarte, CA, 91010.

**A**

**B**

**C**

**CS1-APC**

**Isotype**

**U266B**

**MM.1S**

**MM.1S-CS1^KO^**

**Supplementary Figure 1:** A) CS1 expression levels on multiple myeloma lines MM.1S and U266B. Expression levels were determined following the knockdown of CS1 on the MM.1S line (MM.1S-CS1^KO^). B) Comparison of cell viability during culture of MM.1S and MM.1S-CS1^KO^ lines. C) Cytokine secretion in culture supernatant following co-culture of CS1-dbBiTE coated T cells with MM.1S lines (n=3).


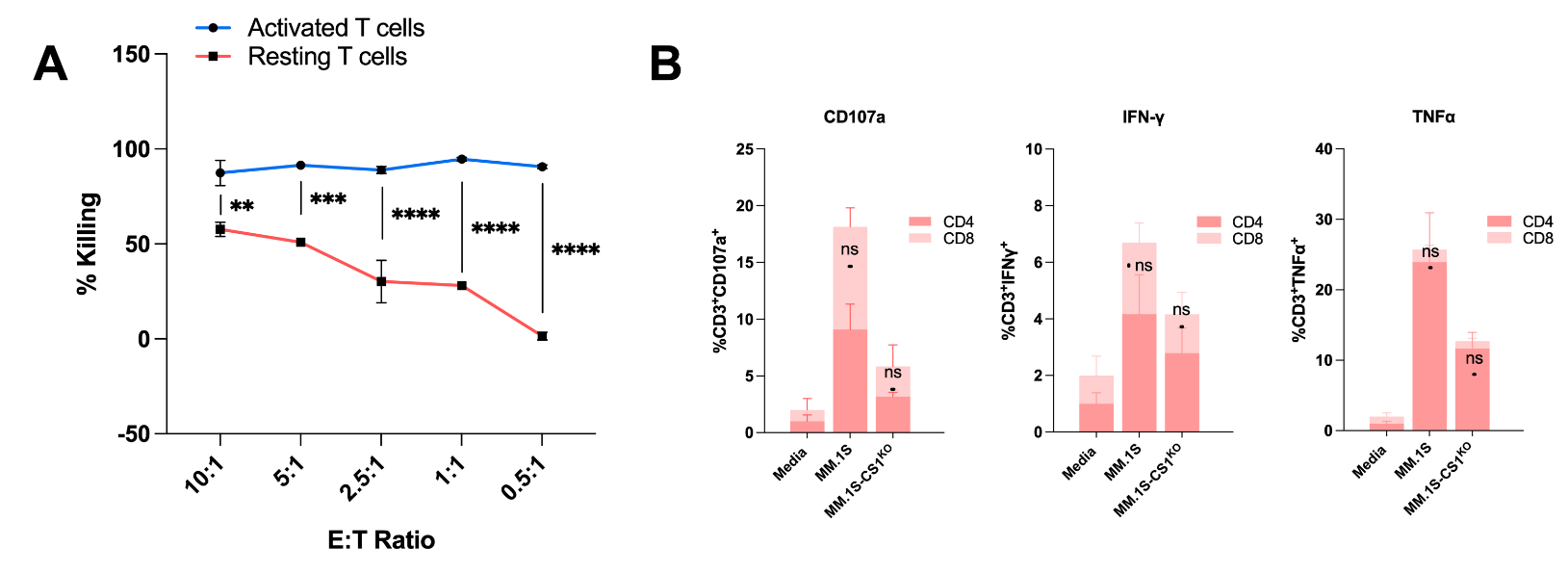


**Supplementary Figure 2:** A) Comparison of MM.1S tumor killing between activated and resting T cells following CS1-dbBiTE coating. Increasing effector target (e:t) ratios are depicted. B) Comparison of CD4 vs CD8 resting T cell subsets during degranulation (CD107) and intracellular cytokine secretion (IFN-γ and TNF-α) following co-culture of coated T cells with respective targets. Data is from 3 independent donors (n=3).


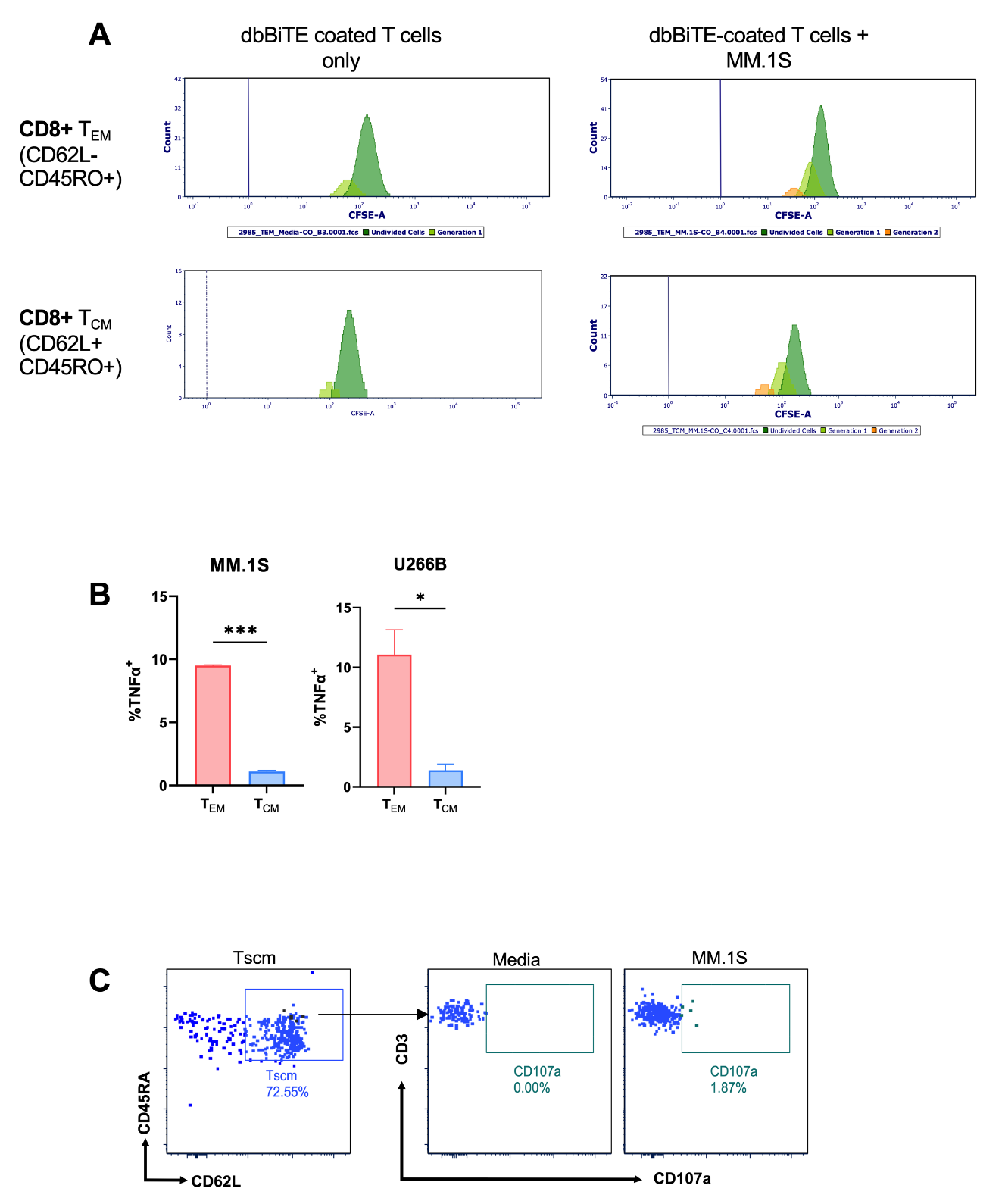


**Supplementary Figure 3:** A) CD8^+^ T cell subset proliferation following CS1-dbBiTE coating alone and in the presence of MM.1S targets. B) Comparison of TNF-α positive cells between effector and central memory T cells after co-culture of CS1-dbBiTE coated T cells from patient samples with MM lines MM.1S and U266B. Data from 2 donors are depicted. C) Expression of CD107a in dbBiTE coated CD45RA+CD62L+ Tscm cells after co-culture with MM.1S.

**CD3**

**CD107a**

**CD3**

**Dapi**

**FSC-A**

**SCC-A**

**FSC-A**

**SCC-A**

**CD3**

**IFNγ**

**CD3**

**TNF-α**

**Dapi**

**TNFα-FMO**

**IFNγ-FMO**

**CD3**

**FSC-A**

**SCC-A**

**Dapi**

**CD3**

**PBMC Sort**

**CD62L**

**CD45RA**

**ICS Gating**

**FSC-A**

**FSC-H**

**FSC-A**

**FSC-H**

**FSC-A**

**FSC-H**

**CD107a Gating**

**Supplementary Figure 4:** Flow cytometry gating strategy used for sample analysis.
